# The Long-Term Socioeconomic Outcomes of Youth Caregivers: Evidence From the Health and Retirement Study

**DOI:** 10.1093/geroni/igaf122.921

**Published:** 2025-12-31

**Authors:** Katherine Miller, Elise Parrish, Yiqing Qian, Regina Shih

**Affiliations:** Johns Hopkins Bloomberg School of Public Health, Baltimore, Maryland, United States; University of Pennsylvania, Philadelphia, Pennsylvania, United States; Johns Hopkins University School of Nursing, Baltimore, Maryland, United States; Emory University Rollins School of Public Health, Atlanta, Georgia, United States

## Abstract

Family (unpaid) caregivers are the backbone of the long-term care delivery system in the United States. The economic consequences of providing unpaid care are overwhelming negative. However, the existing evidence excludes youth family caregivers. Youth caregivers typically have lower educational attainment and worse mental and physical health outcomes than non-caregiving peers, yet the long-term economic outcomes for youth caregiving are unknown. We describe the associations between youth caregiving (aged <25 at time of first caregiving episode) and later-life economic and caregiving outcomes compared to individuals who never report caregiving in their lifetime. We use the Health and Retirement Study Core and Leave Behind Supplement. Among youth caregivers, respondents reported two caregiving episodes (providing unpaid care for ≥6 months) on average over the life course and 15% became a caregiver again after age 50. Compared to individuals who never became caregivers, youth caregivers are significantly more likely to be female (77% vs 51%); have lower levels of life-time educational attainment (25% have bachelor’s or higher versus 34%); and were less likely to initiate post-secondary education within 2 years of graduating high school (-14.2 percentage points). Youth caregivers’ predicted social security value at age 50 is approximately $23,000 less than never caregivers ($75,071 vs. $98,271). Youth caregiving before age 25 is associated with later life socioeconomic status and likelihood of becoming a caregiver in later life. Further research to disentangle the causal effects of caregiving across the life course is necessary to inform targeted policy supports to mitigate long-term adverse economic consequences.

